# Association between glaucoma and risk of stroke: A systematic review and meta-analysis

**DOI:** 10.3389/fneur.2022.1034976

**Published:** 2023-01-13

**Authors:** Meng Wang, Ni Chen, Bai-chao Sun, Chun-Bao Guo, Shuang Zhang, Ming-Jie Huang, Ben-Gang Zhou, Xiang-yu Wang, Zheng-Biao Huang

**Affiliations:** ^1^Department of Neurology, The Third Clinical Medical College of China, Three Gorges University, Gezhouba Central Hospital of Sinopharm, Yichang, China; ^2^Department of Ophthalmology, The Third Clinical Medical College of China, Three Gorges University, Gezhouba Central Hospital of Sinopharm, Yichang, China; ^3^Department of Gastroenterology, The First People's Hospital of Yichang, The People's Hospital of China Three Gorges University, Yichang, China

**Keywords:** glaucoma, stroke, risk, meta-analysis, systematic review

## Abstract

**Background/objectives:**

Recently, several studies explored the association between glaucoma and the risk of stroke, but these results were inconsistent. Therefore, we conducted a meta-analysis to examine this possible association.

**Methods:**

We conducted a systematic literature search of PubMed, Embase, and Web of Science from inception until February 28, 2022. Random-effects meta-analysis was conducted by generic inverse variance method. Sensitivity and subgroup analyses were performed. The review protocol has been registered with PROSPERO (CRD42022312797).

**Results:**

Seven studies (involving 362,267 participants) have been published from 2004 to 2017 and included in the meta-analysis. These studies included four retrospective cohort studies, two cross-sectional studies, and one case–control study. Meta-analysis of these data has shown that glaucoma was associated with an increased risk of stroke (OR = 1.94, 95% CI = 1.45–2.59). Most of the subgroup analyses demonstrated similar results. These findings were stable in sensitivity analyses.

**Conclusions:**

We found that glaucoma was associated with an increased risk of stroke. The result suggests that patients with glaucoma need to be assessed the risk of stroke to reduce the incidence of stroke. To better explore the nature of any association, prospective studies that consider the stroke subtypes, sample size, district, and other confounding factors are needed.

## 1. Introduction

Glaucoma represents a chronic degenerative optic neuropathy characterized by the dysfunction and loss of retinal ganglion cells (RGCs), progressive degeneration of retinal nerve fiber layer, and cupping of the optic disc. Glaucoma can eventually lead to visual field defects (1–3).

Nowadays, glaucoma is the leading cause of global irreversible blindness, some studies reported that approximately 21 million people afflicted by glaucoma in China in 2020, the prevalence among the middle-aged and elderly in Europe was about 2.93%, and due to the aging population, the number of people with glaucoma will increase to 111.8 million worldwide in 2040 (4–6). Nevertheless, glaucoma is not an independent disease, many studies have shown that glaucoma was associated with vascular (7, 8), endocrine (9, 10), neurological (11), and psychological diseases (12).

Stroke is the main cause of death and disability (13), and its pathogenesis includes various factors such as hypertension, genetics, and lifestyle. The age-standardized mortality rates of stroke have significantly decreased from 1990 to 2016, but the age-standardized incidence did not show the same trend, the global burden of stroke, as a result of the aging population, is expected to remain high (14). Therefore, it is necessary to further explore the possible risk factors of stroke.

In recent years, the relationship between glaucoma and stroke has attracted considerable attention. However, the evidence from observational studies (15–23) shows conflicting results between glaucoma and the risk of stroke. Therefore, we conducted a systematic review and meta-analysis of the published observational studies to explore the association of glaucoma with the risk of stroke.

## 2. Methods

### 2.1. Protocol and registration

This systematic review and meta-analysis has been conducted in accordance with the Preferred Reporting Items for Systematic Reviews and Meta-Analysis (PRISMA) statement (24), and this study was registered with the International Prospective Register of Systematic Reviews (PROSPERO) (registration number: CRD42022312797).

### 2.2. Search strategy

We conducted a systematic literature search of PubMed, Embase, and Web of Science. The retrieval time was from database inception to February 2022 to identify observational studies on the association between glaucoma and the risk of stroke, and the reference lists of relevant articles were examined to supplement the search. We used Medical Subject Heading terms in combination with free terms searching, without language restrictions. The detailed search strategies were as follows: (glaucoma OR intraocular pressure OR ocular hypertension OR ocular tension OR intraocular tension OR eye pressure OR eye tension OR intraocular pressure OR intraocular hypertension OR eye ball pressure OR eye internal pressure OR eyeball pressure OR ocular pressure OR eye internal pressure) AND (stroke OR cerebral infarction OR brain infarction OR cerebral hemorrhage OR intracerebral hemorrhage OR transient ischemic attack OR cerebrovascular disorders OR cerebrovascular disorders OR cerebrovascular accident). The full search strategy for PubMed is described in online Supplementary Table 1.

### 2.3. Study selection

The following inclusion criteria were applied: (1) Observational studies (cross-sectional, cohort study, case–control study) investigating the association between glaucoma and the risk of stroke, (2) Studies providing unadjusted or adjusted effect estimates, the odds ratio (OR), risk ratio (RR), and hazard ratio (HR) with corresponding 95% confidence interval (CI), if these data were not provided, then they will be calculated from raw data wherever possible, (3) The included sample size has a clear time period. Excluded criteria were as follows: (1) Abstract, systematic reviews, editorials commentaries, protocols, opinion papers, letters, and reports, (2) No comparison group, and (3) Studies without sufficient data.

Title and abstract screening were done independently by two reviewers for potential eligibility and assessed full-text articles for final eligibility. Discrepancies about selection were resolved through consultation with a third reviewer.

### 2.4. Data extraction

The following variables were abstracted from each study: the first author, publication year, study country, study design, type of glaucoma, type of stroke, study subjects, sample size, mean age or age group, percentage of male, follow-up time or study period, OR, RR, HR (adjusted and unadjusted) with their 95% CI, and adjusted confounding variables. Two reviewers independently compared the data selected and resolved the disagreement through consultation.

### 2.5. Assessment of quality

Two reviewers independently used the Newcastle–Ottawa Scale (NOS) (25) to assess the methodological quality of case–control and cohort studies, the NOS assigns a maximum of 4 points for selection, 2 points for comparability, and 3 points for exposure or outcome. Studies of low-, moderate-, and high-quality studies were defined as NOS scores of 1–3, 4–6, and 7–9, respectively. Whereas, the Agency for Healthcare Research and Quality (AHRQ) (26) checklist was used to evaluate the quality of cross-sectional studies, with a score ranging from 0 to 11. The AHRQ scores of 4–7 and 8–11 indicated moderate and high quality, respectively (27).

### 2.6. Statistical analysis

The meta-analyses were performed using the Review Manager software (Version 5.3), and the odds ratios (ORs) and 95% confidence intervals (CIs) were calculated to evaluate the association between glaucoma and stroke. A random-effect, generic inverse variance method of DerSimonian and Laird was used to estimate the pooled OR and 95% CI. As the outcome of interest was relatively uncommon, we considered RR/HR equivalent to OR (28). When both unadjusted and adjusted OR/HR/RR were reported in a study, the adjusted one was selected. The Cochrane's *Q*-test was performed to evaluate the heterogeneity between studies, *P* < 0.10 for the *Q*-test was considered statistically significant, and an I-squared statistical test was conducted to assess the degree of heterogeneity, *I*^2^ = 0% indicated no heterogeneity, 25–50% for low, 50–75% for moderate, and more than 75% for high heterogeneity (29). Statistical significance was defined as *P*-value < 0.05. Subgroup analyses were performed according to the study design, adjustment for confounders, type of glaucoma, and stroke. Moreover, we performed the sensitivity analysis by deleting each study individually to assess the quality and consistency of the results. Publication bias and meta-regression were not conducted if the small number of studies < 10 was included in this analysis.

## 3. Results

### 3.1. Selection

**Figure 1:**
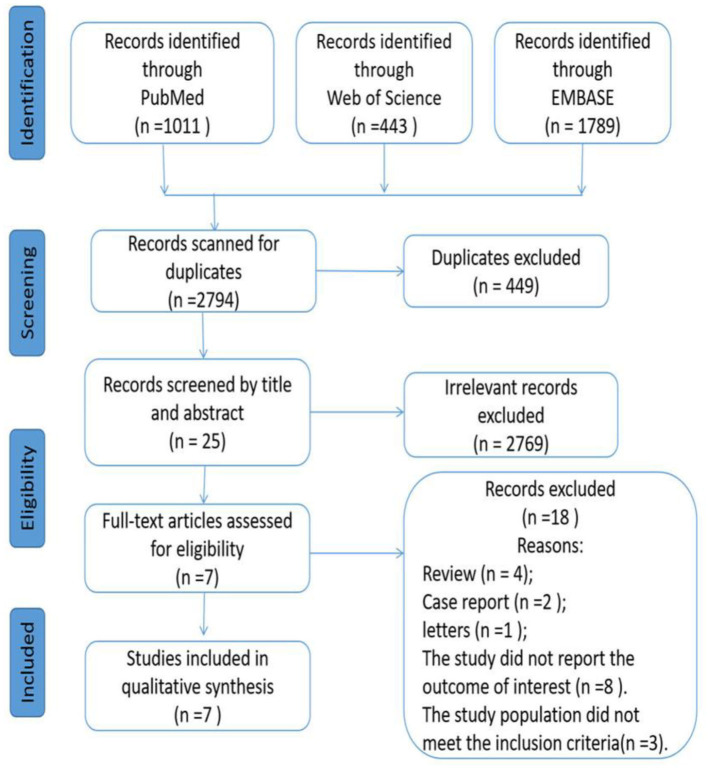
PRISMA flowchart of study selection process.

According to our initial search strategies, 3,243 potentially relevant articles were identified from three electronic databases, of which 449 were excluded as they were duplicates. A total of 2,769 records were excluded after scanning these titles and abstracts. After removing 18 records based on the full-text reading (details of excluded articles see Supplementary Table 2), 7 articles (15, 17–21, 23) met our selection criteria and were included in the study. Figure 1 describes the process of literature screening.

### 3.2. Study characteristics

Table 1 summarizes the main characteristics of the included studies. Seven studies (involving 362,267 participants) have been published from 2004 to 2017. Those studies included four retrospective cohort studies (17, 18, 21, 23), two cross-sectional studies (15, 19), and one case–control study (20). Overall, these studies were conducted in different countries across two continents: six studies (17–21, 23) were conducted in Asia and one study in Europe (15). Sample size among the included studies varied widely, ranging from 100 to 306,692 participants. Regarding the type of glaucoma, five of the seven studies (15, 17, 19–21) included open-angle glaucoma, and two studies (18, 23) involved neovascular glaucoma and normal-tension glaucoma, respectively. Regarding the type of stroke, four studies (17–19, 21) did not define the subtypes of stroke, two studies (20, 23) contain different subtypes (ischemic stroke, hemorrhagic stroke), and one study (15) involved ischemic stroke. The following data were extracted from each included study (details of information see Supplementary Table 3). Quality of five studies (17, 18, 20, 21, 23) was assessed with the NOS (NOS scores ranged from 6 to 7), two studies (15, 19) were assessed with the AHRQ (AHRQ scores ranging from 5 to 8) (see Supplementary Table 4).

**Table 1:** Main characteristics of included studies.

**References**	**District**	**Study design**	**Type of glaucoma**	**Type of stroke**	**Sample size**	**Mean age or Age group (years), male (%)**	**Stroke verification**	**NOS/AHRQ score**
Su (23)	Taiwan	Retrospective cohort	Neovascular glaucoma	Ischemic stroke Hemorrhagic stroke	4,576	61,57.9%	Medical records	7
Rim (21)	Korea	Retrospective cohort	Open angle glaucoma	Unspecific stroke	9,090	< 65; ≥65, 53.84%	Medical records	7
Lee (18)	Taiwan	Retrospective cohort	Normal-tension glaucoma	Unspecific stroke	5,658	54,51.43%	Medical records	7
Lin (20)	Taiwan	A case-control	Open-angle glaucoma	Ischemic infarction, intracerebral hemorrhage, subarachnoid hemorrhage, and transientischemic attack	306,692	63,52.21%	Medical records	6
Ho (17)	Taiwan	Retrospective cohor	Open-angle glaucoma	Unspecific stroke	24,192	60,49.20%	Medical records	7
Lee (19)	Korea	Cross-sectional	Open-angle glaucoma	Unspecific stroke	11,959	(40–49, 50–59, 60–69, 70–79 and >80), 47.8%	Medical records	8
Belzunce (15)	Spain	Cross-sectional	Primary open angle glaucoma	Ischemic stroke	100	71,48%	Medical records	5

NVG, Neovascular glaucoma; NTG, Normal-tension glaucoma; OAG, Open-Angle Glaucoma; POAG, Primary open angle glaucoma; NOS, Newcastle-Ottawa Scale; NR, not reported.

### 3.3. Association between glaucoma and risk of stroke

**Figure 2:**
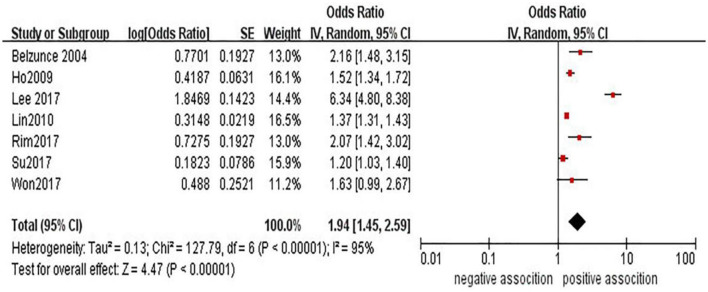
Forest plot of evaluating the association between glaucoma and stroke.

The association between glaucoma and the risk of stroke has been demonstrated in seven studies (15, 17–21, 23). In a pooled analysis of these seven studies, glaucoma was associated with stroke (OR = 1.94, 95% CI = 1.45–2.59) (see Figure 2). However, high heterogeneity (*I*^2^ = 95%, *P* < 0.00001) was observed in the analysis, and sensitivity analyses indicated that the study from Lee et al. (18) was mainly responsible for the observed heterogeneity (see Supplementary Table 5).

To explain the high heterogeneity, several subgroup analyses were performed. Details of all subgroup analyses are provided (see Table 2; Supplementary Figures 1–8). On subgroup analysis by study design, we found a significant positive association between glaucoma and risk of stroke among four retrospective cohort studies (17, 18, 21, 23) (*n* = 4, OR = 2.19; 95% CI = 1.21–3.97; *P* = 0.01; *I*^2^ = 97%), two cross-sectional studies (15, 19) (*n* = 2, OR = 1.95; 95% CI = 1.44–2.63; *P* < 0.0001; *I*^2^ = 0%), and one case–control study (20) (*n* = 1, OR = 1.37; 95% CI = 1.31–2.63; *P* < 0.0001). In the subgroup analysis stratified by type of glaucoma, we found a significant positive association between open-angle glaucoma and stroke (*n* = 5, OR = 1.43; 95% CI = 1.27–1.61; *P* < 0.00001; *I*^2^ = 65%), normal-tension glaucoma and stroke (*n* = 1, OR = 6.34; 95% CI = 4.80–8.38; *P* < 0.00001), and neovascular glaucoma and stroke (*n* = 1, OR = 2.07; 95% CI = 1.42–3.02; *P* = 0.0002). In the subgroup analysis stratified by type of stroke, we found a significant positive association between glaucoma and unspecific stroke (*n* = 5, OR = 1.88; 95% CI = 1.34–2.66; *P* = 0.0003; *I*^2^ = 97%) and ischemic stroke (*n* = 2, OR = 2.20; 95% CI = 1.67–2.89; *P* < 0.00001; *I*^2^ = 0); however, no significance was observed for study with hemorrhagic stroke (*n* = 1, OR = 1.15; 95% CI = 0.35–3.78; *P* = 0.82). As we all know, gender, age, and region are also risk factors for cerebrovascular disease. We continued to conduct the subgroup analysis of patients stratified by these three variables. In the subgroup analysis stratified by adjustment for confounders, we found a significant positive association between glaucoma and stroke with adjusted OR (*n* = 6, OR = 1.91; 95% CI = 1.40–2.61; *P* < 0.0001; *I*^2^ = 96%) and crude OR (*n* = 5, OR = 2.44; 95% CI = 1.68–3.56; *P* < 0.00001; *I*^2^ = 98%), respectively. In subgroup analysis stratified by mean age, we found a significant positive association between glaucoma and risk of stroke under 65 years (*n* = 5, OR = 1.95; 95% CI = 1.34–2.86; *P* = 0.0006), no significance was observed for the study with more than or equal to 65 years old (*n* = 2, OR = 1.59; 95% CI = 0.92–2.75; *P* = 0.1). The results of the subgroup analyses based on gender ratio of study participants, study region, and study quality were consistent with the overall pooled results.

**Table 2:** Subgroup analyses of association between glaucoma and risk of stroke.

**Subgroup**	**No. of studies**	**OR (95%CI)**	***P*_association_**	***I^2^* (%)**	***P*_heterogeneity_**
Overall studies	7	1.94 (1.45–2.59)	< 0.00001	95	< 0.00001
**Study design**					
Retrospective cohort	4	2.19 (1.21–3.97)	0.01	97	< 0.00001
Case-control	1	1.37 (1.31–1.43)	< 0.00001	–	–
Cross-sectional	2	1.95 (1.44–2.63)	< 0.0001	0	0.37
**Type of stroke**					
Unspecific stroke	5	1.88 (1.34–2.66)	0.0003	97	< 0.00001
Ischemic stroke	2	2.20 (1.67–2.89)	< 0.00001	0	0.90
Hemorrhagic stroke	1	1.15 (0.35–3.78)	0.82	–	–
**Type of glaucoma**					
Open-angle glaucoma	5	1.43 (1.27–1.61)	< 0.00001	65	0.02
Normal-tension glaucoma	1	6.34 (4.80–8.38)	< 0.00001	–	–
Neovascular glaucoma	1	2.07 (1.42–3.02)	0.0002	–	–
**Adjustment for confounders**					
Adjusted	6	1.91 (1.40–2.61)	< 0.0001	96	< 0.00001
Unadjusted	5	2.44 (1.68–3.56)	< 0.00001	98	< 0.00001
**Study quality**					
High quality	5	2.08 (1.23–3.49)	0.006	96	< 0.00001
Moderate quality	2	1.65 (1.07–2.56)	0.03	82	0.02
**Age**					
< 65 years	5	1.95 (1.34–2.86)	0.0006	97	< 0.00001
≥ 65 years	2	1.59 (0.92–2.75)	0.1	85	0.009
**Region**					
Asia	6	1.91 (1.40–2.61)	< 0.0001	96	< 0.00001
Europe	1	2.16 (1.48–3.15)	< 0.0001		
**Gender**					
Male > Female	4	2.12 (1.26–3.58)	0.005	98	< 0.00001
Male < Female	3	1.66 (1.35–2.04)	< 0.00001	34	0.22

OR, odds ratio; CI, confidence interval.

In addition, we performed sensitivity analysis to confirm the robustness of the results. After excluding studies one by one from the meta-analysis, these results did not materially change in direction or magnitude (see Supplementary Table 5).

## 4. Discussion

Exploring an association between glaucoma and stroke could be meaningful, for both are common medical disorders, this gives rise to concern from a public health point of view. The purpose of this research was to synthesize the published observational studies on the association between glaucoma and stroke risk through a systematic review and meta-analysis. To the best of our knowledge, the present study is the first meta-analysis on this topic to date. In this meta-analysis of seven studies involving 362,267 participants, we demonstrated that glaucoma was associated with an increased risk of stroke (OR = 1.94, 95% CI: 1.45–2.59, *P* < 0.00001).

Six of the seven articles reported their results after adjusting for confounding factors. Four retrospective cohort studies (17, 18, 21, 23), one cross-sectional study (19), and a case–control study (20) adjusted some comorbidities such as hypertension, diabetes, chronic renal failure, atrial fibrillation, cardiovascular disease, and sociodemographic factors including age, gender, residential area, and household income. After removing the article (15) of unadjusted confounding factors, we also got the same conclusion (OR = 1.91, 95% CI: 1.40–2.61, *P* < 0.00001).

The meta-analysis included observational studies that can only provide details about correlations but not the causality. However, the association between OAG and stroke risk may also be explained by shared risk factors. The mechanism of glaucomatous optic neuropathy is unknown, but ischemic damage plays an important role in glaucomatous optic nerve damage (30). Impaired vascular may cause vascular dysregulation and defective autoregulation of ocular blood flow, especially in OAG and NTG (31, 32), eventually leading to ischemic optic nerve damage and glaucomatous optic neuropathy (33). This kind of vascular change may occur in the brain (34, 35), the ophthalmic artery, which branches of internal carotid artery, branches to form the central retinal artery and the posterior ciliary arteries (PCAs), which is the main blood supply of the optic nerve head (36). Compared with the non OAG, cerebral white matter lesions representing white matter structural damage due to vascular disease was significantly increased (37), in addition to that, fundus photographs show the retinal nerve fiber defects, which were associated with cerebral small vessel disease such as white matter lesions are frequently observed on MRI (38). Several studies have shown that OAG and stroke have associated with vascular disorders, such as the narrowing of retinal arterioles and carotid atherosclerosis (39, 40). Common risk factors for stroke include chronic kidney disease (41), hypertension (42), and diabetes mellitus (43) were also related to the OAG and narrowing of retinal arterioles, which have been found to predict the risk of silent cerebral infarcts and overt stroke (40, 44). Neovascular glaucoma (NVG) is caused by retinal ischemia, which produces the vascular endothelial growth factor (VEGF), some studies findings that the level of VEGF also increases in ischemic stroke, which may share a common mechanism with NVG (45–47). Thus, we suspected that the association between glaucoma and stroke may be due to shared systemic risk factors. However, the precise mechanisms associated with glaucoma and stroke were not fully understood and need to be further studied.

Considering the significant heterogeneity of this study, we plan to use meta-regression and subgroup analysis to explore the source of heterogeneity. Because of the limited number of related studies, we just performed subgroup analyses depending on several methodologic and clinical features. Four retrospective studies (17, 18, 21, 23), two cross-sectional studies (15, 19), and one case–control study (20) show that glaucoma was associated with an increased risk of stroke. However, this conclusion must be treated with caution since the limited number of studies in these subgroups. Regarding the different types of glaucoma, the subgroup analysis shows that five studies (15, 17, 19–21) explored the OAG associated with stroke, and only two articles (18, 23) described the relationship between NVG, NTG, and the risk of stroke, respectively. Due to the lack of data available for analysis of different types of glaucoma in the original articles, caution should be exercised in extrapolating the results, further studies are needed to investigate the association between the different types of glaucoma and the risk of stroke. Due to the different types of strokes, analysis was performed separately for ischemic stroke, hemorrhagic stroke, and unspecific stroke. Our subgroup analysis showed that glaucoma was associated with ischemic and unspecific stroke but not with hemorrhagic stroke. The reason for this result may be that five of the seven articles do not distinguish the type of stroke, resulting in insufficient data for detailed analysis. Hence, further research is needed to detect the relationship between glaucoma and the risk of specific stroke types. In the subgroup analysis that depends on different study locations, we found a significant association between glaucoma and stroke in Asia (17–21, 23) and Europe (15). However, the credibility of the conclusion drawn is not high in this study. In our research, just six studies were conducted in Asia (one study in Korea and five studies in Taiwan), and one study was conducted in Europe, this conclusion was strictly restricted by region. As a result of aging people, the incidence rate of glaucoma and stroke is increased. So, we speculate that the risk of stroke in glaucoma should show higher risk among men and women who over 65 years old. Interestingly, we concluded that there was a significant association between glaucoma and the risk of stroke in people under 65 years old, but this conclusion not happened in people over 65 years old, which was contrary to our expectations. A possible explanation for these opposite results is that studies on this subject were limited by sample sizes and number of studies, especially in this subgroup, making the results less reliable.

Sensitivity analysis and most of the results of the subgroup analysis suggest that these meta-analysis results were not affected by the included study or study characteristics. Thus, this suggested that the results of the meta-analysis were relatively stable and statistically robust. However, this result needs to be treated with caution. Sensitivity analysis revealed no change in the results. But after removing the study by Lee et al., the degree of heterogeneity decreased from high to medium, with the *I*^2^ index decreasing from 96 to 68%, revealing it as the source of heterogeneity. This difference may be caused by the following reasons: the average age (54 years old) of this article was little younger than that in other articles (62 years old), in addition to that, this article studied the relationship between normal-tension glaucoma (NTG) and stroke but did not provide a clear type of stroke. Meanwhile, other potential sources of heterogeneity may exist that we failed to identify and test due to the overall small number of studies.

This study has some strengths and limitations that are worth considering. First, owing to the high prevalence and incidence of both glaucoma and stroke globally, the topic of our study is meaningful and clinically relevant. To our knowledge, this is the first meta-analysis to date to explore the association between glaucoma and the risk of stroke with a large sample size [362,267], we believe that this systematic review and meta-analysis provides the comprehensive assessment of the association between glaucoma and risk of stroke. Second, we made a broad, strict search strategy and conducted independent screening, full-text review, and extraction by two reviewers to ensure the accuracy and integrity of data. In addition to that, in order not to omit studies that meet the inclusion criteria, “Rey” literature in Embase and Web of Science databases was also searched, and we try our hardest to exclude the low-quality studies by using stringent inclusion criteria. Third, we performed subgroup analysis and rigorous sensitivity analysis to evaluate the association between glaucoma and the risk of stroke. Together these results suggest that our conclusion is reliable and convincing.

The results of this meta-analysis are new, but some potential limitations need to be pointed out. First, all studies that we included are observational studies that the confounding factors might be present in the original studies, and this may reduce the credibility of our results. Several cerebrovascular disease risk factors, such as gender, age, hypertension, diabetes, and atrial fibrillation, have been adjusted in most articles. However, potential confounding variables, such as smoking, drinking, body mass index, cerebrovascular disease family history, and other cerebrovascular risk factors, were not adjusted. Furthermore, prospective studies with appropriate controls that adjust for potential confounders are urgently warranted to further examine the association between glaucoma and the risk of stroke. Second, the results may not generalize to other districts given that the geographic distribution of the included results is limited, with most studies conducted in the Asian region. Third, data for glaucoma, stroke, and other comorbidities were collected from a secondary claims database, inaccurate or incomplete information about these records may cause data inaccuracy, in addition to that, due to lack of sufficient data on the severity of glaucoma, the association between the severity of glaucoma and the risk of stroke is still unclear.

## 5. Conclusion

In summary, we found that glaucoma was associated with an increased risk of stroke. The result suggests that patients with glaucoma need to be assessed the risk of stroke to reduce the incidence of stroke. To better explore the nature of any association, prospective studies that consider the stroke subtypes, sample size, district, and other confounding factors are needed.

## Data availability statement

The original contributions presented in the study are included in the article/Supplementary material, further inquiries can be directed to the corresponding author.

## Author contributions

MW and NC performed the literature search and drafted the manuscript. MW and B-cS screened the literature. NC, C-BG, and SZ extracted data and assessed the quality. M-JH, Z-BH, and X-yW analyzed and interpreted the data. B-GZ and MW designed the systematic review. MW, B-GZ, and NC were responsible for the research design, data analysis, and manuscript revision. All the authors gave their approval for the submission of the final manuscript.
