# Supplementary material for: Association between glaucoma and risk of stroke: A systematic review and meta-analysis
Source: Front Neurol. 2023 Jan 13;13:1034976. doi: 10.3389/fneur.2022.1034976 (PMC9881460; doi:10.3389/fneur.2022.1034976)
Supplement: Supplementary file 1 [file Data_Sheet_1.docx]

| **Supplementary online material** | | |
| --- | --- | --- |
| **Content** | | **Page** |
| **Table S1.** | Representatives search strings for PubMed | 2 |
| **Table S2.** | Description of excluded studies | 3-5 |
| **Table S3.** | The other Characteristics of included studies | 6-7 |
| **Table S4.** | Methodological quality assessment of included studies with NOS and AHRQ | 8 |
| **Table S5.** | Results of sensitivity analyses | 9 |
| **Figure S1**. | Forest plot of subgroup analysis stratified by study design | 10 |
| **Figure S2**. | Forest plot of subgroup analysis stratified by type of stroke | 10 |
| **Figure S3**. | Forest plot of subgroup analysis stratified by type of glaucoma | 11 |
| **Figure S4**. | Forest plot of subgroup analysis stratified by adjustment for confounders | 11 |
| **Figure S5.** | Forest plot of subgroup analysis stratified by study region | 12 |
| **Figure S6.** | Forest plot of subgroup analysis stratified by type of mean age | 12 |
| **Figure S7.** | Forest plot of subgroup analysis stratified by type of gender | 13 |
| **Figure S8.** | Forest plot of subgroup analysis stratified by adjustment for study quality | 13 |

| **Table S1. Representatives search strings for PubMed**  **(From inception to February 2022)** | |
| --- | --- |
| **Databases** | **Search strings** |
| **PubMed** | "Glaucoma"[MeSH Terms] OR glaucoma[tiab]  OR "Intraocular Pressure"[MeSH Terms] OR "intraocular pressure"[tiab]OR "Ocular Hypertension"[MeSH Terms] OR "ocular hypertension"[tiab] OR “ocular tension”[tiab] OR “intraocular tension”[tiab] OR “eye pressure”[tiab] OR “eye tension”[tiab] OR “intraorbital pressure”[tiab] OR “intraocular hypertension”[tiab] OR “eye ball pressure”[tiab] OR “eye internal pressure”[tiab] OR “eyeball pressure”[tiab] OR “ocular pressure”[tiab] OR “eye internal pressure”[tiab] AND ("stroke"[MeSH Terms] OR "stroke"[tiab] OR "cerebral Infarction"[MeSH Terms] OR "cerebral Infarction"[tiab] OR "brain infarction"[MeSH Terms] OR "brain infarction"[tiab] OR "cerebral hemorrhage"[MeSH Terms] OR "cerebral hemorrhage"[tiab] OR "intracerebral hemorrhage"[tiab] OR "transient ischemic attack"[tiab] OR "cerebrovascular disorders"[MeSH Terms] OR "cerebrovascular disorders"[tiab] OR "cerebrovascular accident"[tiab]) |

| **Table S2. Description of excluded studies at the stage of eligibility**  **according to the PRISMA flow chart.** | | | |
| --- | --- | --- | --- |
| **No.** | **First author** | **Publication year** | **Reason for exclusion** |
| 1. | French DD | 2010 | letters |
| 2. | Hadeel Sadek | 2021 | The study did not report the outcome of interest |
| 3. | Leung DY | 2009 | The study did not report the outcome of interest |
| 4. | Chou CC | 2018 | The study population did not meet the inclusion criteria |
| 5. | Andrzej Grzybowski | 2020 | Review |
| 6. | Andrew Huck | 2014 | Review |
| 7. | Muneeb A Faiq | 2019 | The study population did not meet the inclusion criteria |
| 8. | Olubor OJ | 2016 | The study did not report the outcome of interest |
| 9 | Coppeto JR | 1985 | Case Reports |
| 10 | MélikParsadaniantz S | 2020 | Review |
| 11 | Wey S | 2019 | Review |
| 12 | Lee SH | 2017 | The study did not report the outcome of interest |
| 13 | Perera N | 2019 | Case Reports |
| 14 | Güngör IU | 2011 | The study did not report the outcome of interest |
| 15 | Zhokhov VP | 1976 | The study did not report the outcome of interest |
| 16 | Ramrattan RS | 2001 | The study did not report the outcome of interest |
| 17 | Basinskiĭ SN | 1990 | The study did not report the outcome of interest |
| 18 | Vidal KSM | 2020 | The study population did not meet the inclusion criteria |

**References for the table S2**

1. French DD, Margo CE. Open angle glaucoma and stroke. Ophthalmology. 2010 Aug;117(8): 1653.e3-4. doi: 10.1016/j.ophtha.2010.03.016. PMID: 20682379.

1. Hadeel Sadek; Ibraheem S Shaikh; Samer T Elsamna; Albert S Khouri. Assessing open angle glaucoma as an independent risk factor for stroke. Investigative Ophthalmology & Visual Science June 2021, Vol.62, 1621.

1. Leung DY, Tham CC, Li FC, Kwong YY, Chi SC, Lam DS. Silent cerebral infarct and visual field progression in newly diagnosed normal-tension glaucoma: a cohort study. Ophthalmology. 2009 Jul;116(7):1250-6. doi: 10.1016/j.ophtha.2009.02.003. Epub 2009 May 30. PMID: 19481813.

1. Chou CC, Hsu MY, Lin CH, Lin CC, Wang CY, Shen YC, Wang IJ. Risk of developing open-angle glaucoma in patients with carotid artery stenosis: A nationwide cohort study. PLoS One. 2018 Apr 23;13(4): e0194533. Doi: 10.1371/journal.pone.0194533. PMID: 29684030; PMCID: PMC5912741.

1. Grzybowski A, Och M, Kanclerz P, Leffler C, Moraes CG. Primary Open Angle Glaucoma and Vascular Risk Factors: A Review of Population Based Studies from 1990 to 2019. J Clin Med. 2020 Mar 11;9(3):761. doi: 10.3390/jcm9030761. PMID: 32168880; PMCID: PMC7141380.

1. Huck A, Harris A, Siesky B, Kim N, Muchnik M, Kanakamedala P, Amireskandari A, Abrams-Tobe L. Vascular considerations in glaucoma patients of African and European descent. Acta Ophthalmol. 2014 Aug;92(5): e336-40. doi: 10.1111/aos.12354. Epub 2014 Jan 25. PMID: 24460758; PMCID: PMC4107083.

1. Faiq MA, Wollstein G, Schuman JS, Chan KC. Cholinergic nervous system and glaucoma: From basic science to clinical applications. Prog Retin Eye Res. 2019 Sep; 72:100767. doi: 10.1016/j.preteyeres.2019.06.003. Epub 2019 Jun 23. PMID: 31242454; PMCID: PMC6739176.

1. Olubor OJ, Uhumwangho OM, Omoti AE. Ocular disorders in stroke patients in a tertiary hospital in Nigeria. Niger J Clin Pract. 2016 May-Jun;19(3):397-400. doi: 10.4103/1119-3077.179290. PMID: 27022807.

1. Coppeto JR, Wand M, Bear L, Sciarra R. Neovascular glaucoma and carotid artery obstructive disease. Am J Ophthalmol. 1985 May 15;99(5):567-70. doi: 10.1016/s0002-9394(14)77960-7. PMID: 2408476.

1. Mélik Parsadaniantz S, Réaux-le Goazigo A, Sapienza A, Habas C, Baudouin C. Glaucoma: A Degenerative Optic Neuropathy Related to Neuroinflammation? Cells. 2020 Feb 25;9(3):535. doi: 10.3390/cells9030535. PMID: 32106630; PMCID: PMC7140467.

**11**. Wey S, Amanullah S, Spaeth GL, Ustaoglu M, Rahmatnejad K, Katz LJ. Is primary open-angle glaucoma an ocular manifestation of systemic disease? Graefes Arch Clin Exp Ophthalmol. 2019 Apr;257(4):665-673. doi: 10.1007/s00417-019-04239-9. Epub 2019 Jan 15. PMID: 30643967.

**12**. Lee SH, Kim GA, Lee W, Bae HW, Seong GJ, Kim CY. Vascular and metabolic comorbidities in open-angle glaucoma with low- and high-teen intraocular pressure: a cross-sectional study from South Korea. Acta Ophthalmol. 2017 Nov;95(7): e564-e574. doi: 10.1111/aos.13487. Epub 2017 Jul 5. PMID: 28677865.

1. Perera N, Shields M, Perera M, Adler PA. When 'glaucomatous fields' are not glaucoma: bilateral calcarine fissure strokes masquerading as glaucoma in a normal tension glaucoma suspect. BMJ Case Rep. 2019 Mar 21;12(3): e227803. doi: 10.1136/bcr-2018-227803. PMID: 30902842; PMCID: PMC6453379.

1. Güngör IU, Güngör L, Ozarslan Y, Arıtürk N, Beden U, Erkan D, Onar MK, Oge I. Is symptomatic atherosclerotic cerebrovascular disease a risk factor for normal-tension glaucoma? Med Princ Pract. 2011;20(3):220-4. doi: 10.1159/000323596. Epub 2011 Mar 29. PMID: 21454990.

1. Zhokhov VP, Indeĭkin EN, Mitropol'skiĭ AI. O vliianii sezonnykh faktorov na chastotu vozniknoveniia ostrykh pristupov pervichnoĭ glaukomy, gipertonicheskikh krizov i insul'tov [Effect of seasonal factors on the incidence of acute attacks of primary glaucoma, hypertensive crises and strokes]. Vestn Oftalmol. 1976;(1):7. Russian. PMID: 130711

1. Ramrattan RS, Wolfs RC, Panda-Jonas S, Jonas JB, Bakker D, Pols HA, Hofman A, de Jong PT. Prevalence and causes of visual field loss in the elderly and associations with impairment in daily functioning: the Rotterdam Study. Arch Ophthalmol. 2001 Dec;119(12):1788-94. doi: 10.1001/archopht.119.12.1788. Erratum in: Arch Ophthalmol 2002 Apr;120(4):525. PMID: 11735788.

1. Basinskiĭ SN, Sas'ko VI, Matveev EV, Kiridon AG. Korreliatsiia pokazateleĭ tsentral'noĭ i regionarnoĭ gemodinamiki glaza u bol'nykh otkrytougol'noĭ glaukomoĭ [Correlation of the parameters of central and regional hemodynamics of the eye in patients with open-angle glaucoma]. Vestn Oftalmol. 1990 May-Jun;106(3):33-5. Russian. PMID: 2385900.

1. Vidal KSM, Bertola L, Suemoto CK, Moreno AB, Duncan B, Schmidt MI, Maestri M, Barreto SM, Lotufo PA, Benseñor IM, Brunoni AR. Glaucoma, but not cataracts, predicts lower verbal fluency performance: 3.8-year follow-up from the ELSA-Brasil study. Neuropsychol Dev Cogn B Aging Neuropsychol Cogn. 2021 Nov;28(6):871-883. doi: 10.1080/13825585.2020.1837723. Epub 2020 Oct 19. PMID: 33073671.

| **Table S3 The Characteristics of included studies about continents, study subjects, follow-up time (years)/study period,**  **confounders adjustment and corresponding data in the meta-analysis** | | | | | |
| --- | --- | --- | --- | --- | --- |
| **First author, year** | **Continents** | **Study subjects** | **Follow-up time (years)/**  **Study period** | **Confounders adjustment** | **OR/HR (95%CI)** |
| Su,2017 | Asia | Subjects with the Longitudinal Health Insurance Research Database 2000 (LHID2000) | The mean follow-up periods were 4.17 and 5.21 years for the NVG and control cohorts, respectively/January 1,2000 to December 31,2011 | Sex, age, medical comorbidities (DM, hypertension, hyperlipidemia, coronary artery disease) and ocular comorbidities (CRVO/BRVO, DMRP, retinal detachment with retinal defect, HTR, uveitis) | aHR:  All: 2.07（1.41-3.02）  HS:1.15(0.35-3.85)  IS:2.24(1.51-3.32) |
|  |  |  |  |  |  |
| Rim,2017 | Asia | Subjects with the Korean National Health Insurance Service (KNHIS) | The follow-up period was10-year /January 2004 to December 2007 | Comorbidities (HTN, DM, chronic renal failure, AF, and hyperlipidemias) and sociodemographic factors (age, gender, residential area and household income) | aHR:  1.20 (1.03-1.40) |
|  |  |  |  |  |  |
| Lee,2017 | Asia | Subjects with the National Health Insurance Research Database (NHIRD) | The follow-up period was10-year /January 1, 2001 to December 31, 2010 | Age, gender, HTN, diabetes, CHF, IHD, AF, and disorders of lipid metabolism | aHR:  6.34 (4.80–8.38) |
|  |  |  |  |  |  |
| Lin,2010 | Asia | Subjects with the National Health Insurance Research Database (NHIRD) | 2005 | Gender, age, monthly income, and level of urbanization of the patient’s community | aOR:  1.37(1.32-1.43) |
|  |  |  |  |  |  |
| Ho，2009 | Asia | Subjects with the National Health Insurance Research Database (NHIRD) | The follow-up period was5-year/January 1, 2001 to December 31, 2001 | Gender, age, hypertension, diabetes, hyperlipidemia, CHD, and geographic region | aHR:  1.52 (1.40–1.72) |
|  |  |  |  |  |  |
| Won,2017 | Asia | Subjects with the Korea National Health and Nutrition Examination Survey (KNHANES) | 2010-2012 | Gender, age, hypertension, diabetes, dyslipidemia, coronary arterial disease, BMI and smoking | aOR:  1.629(0.994-2.670) |
|  |  |  |  |  |  |
| Belzunce,2004 | Europe | Subjects with the Notre Dame Road Hospital (NDRH) | 2000 | None | cOR:2.16(1.01-2.02) |

| DM, diabetes mellitus; NVG, Neovascular Glaucoma; HS, Hemorrhagic stroke; CRVO/BRVO, central and branch retinal vein occlusion; DMRP, diabetic retinopathy; IS: Ischemic stroke;HTN,hypertension;AF,atrial fibrillation;CHF,congestive heart failure;IHD,ischemic heart disease;CHD,coronary heart disease;BMI,Body Mass Index;HTR,hypertensive retinopathy;aHR:adjusted HR;aOR, adjusted OR;cOR:crude OR;OR, odds ratio; HR, hazard ratio; CI, confidence interval |
| --- |

| **Table S4 Methodological quality assessment of included studies with NOS.** | | | | | | | | | | | | | | | | | |
| --- | --- | --- | --- | --- | --- | --- | --- | --- | --- | --- | --- | --- | --- | --- | --- | --- | --- |
| First, author | | | Selection | | | Comparability | | Exposure/Outcome | | | | | | Total (0-9) | | Quality | |
| Su,2017 | | | 🟑🟑🟑🟑 | | | 🟑🟑 | | 🟑🟑 | | | | | | 7 | | High | |
| Rim,2017 | | | 🟑🟑🟑🟑 | | | 🟑🟑 | | 🟑🟑 | | | | | | 7 | | High | |
| Lee,2017 | | | 🟑🟑🟑🟑 | | | 🟑🟑 | | 🟑🟑 | | | | | | 7 | | High | |
| Lin,2010 | | | 🟑🟑 | | | 🟑🟑 | | 🟑🟑 | | | | | | 6 | | Moderate | |
| Ho，2009 | | | 🟑🟑🟑🟑 | | | 🟑🟑 | | 🟑🟑 | | | | | | 7 | | High | |
| **Methodological quality assessment of included studies with AHRQ.** | | | | | | | | | | | | | | | | | |
| First, author | 1 | 2 | | 3 | 4 | | 5 | 6 | 7 | 8 | 9 | 10 | 11 | | Total (0-9) | | Quality |
| Belzunce,2004 | Y | Y | | Y | U | | U | Y | U | U | U | Y | N | | 5 | | Moderate |
| Won,2017 | Y | Y | | Y | Y | | N | Y | Y | Y | U | Y | N | | 8 | | High |
| Note: NOS, Newcastle–Ottawa Scale. AHRQ, The Agency for Healthcare Research and Quality Y: yes, N:no, U: unclear | | | | | | | | | | | | | | | | | |

| **Table S5 Results of sensitivity analyses** | | | |
| --- | --- | --- | --- |
| **Studies omitted** | **OR (95% CI)** | ***P*association** | **Heterogeneity** |
| Belzunce,2004 | 1.91(1.40-2.61) | P＜0.0001 | I^2^=96% P＜0.00001 |
| Ho,2009 | 2.05(1.36-3.08) | P=0.0006 | I^2^=96% P＜0.00001 |
| Lee,2017 | 1.48(1.31-1.68) | P＜0.00001 | I^2^=68% P=0.007 |
| Lin,2010 | 2.09(1.32-3.29) | P=0.002 | I^2^=95% P＜0.00001 |
| Rim,2017 | 2.13(1.48-3.06) | P＜0.0001 | I^2^=96% P＜0.00001 |
| Su,2017 | 1.92(1.40-2.63) | P＜0.0001 | I^2^=96% P＜0.00001 |
| Won,2017 | 1.99(1.45-2.72) | P＜0.0001 | I^2^=96% P＜0.00001 |


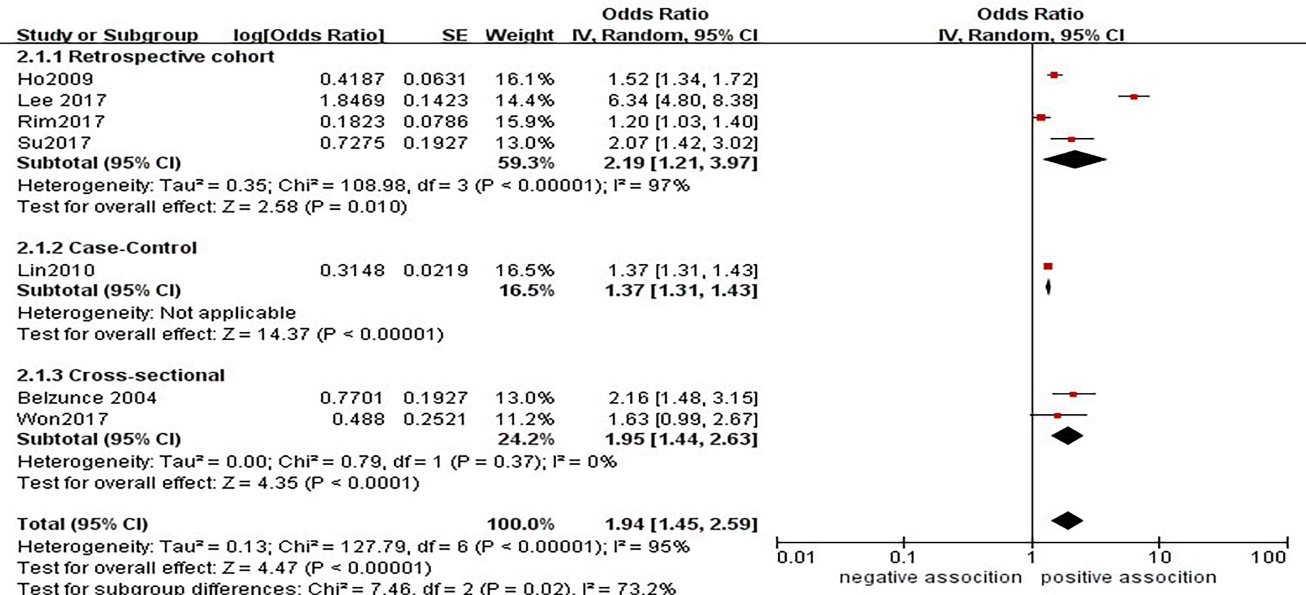


**Figure S1**. Forest plot of subgroup analysis stratified by study design


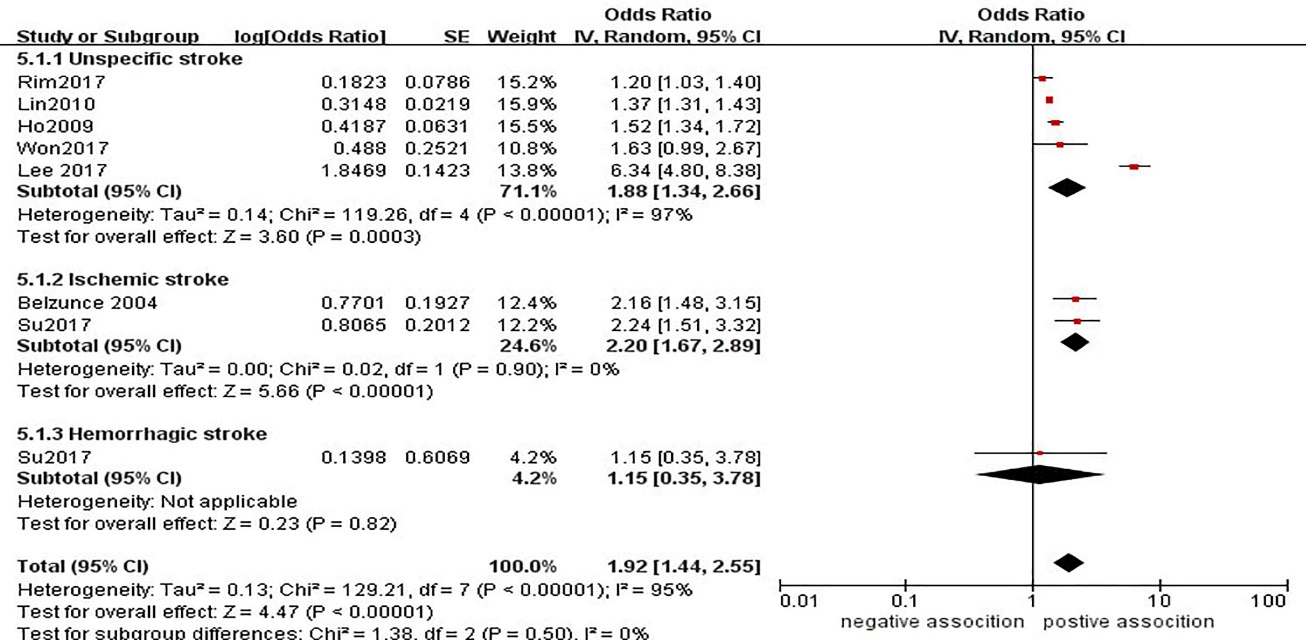


**Figure S2** Forest plot of subgroup analysis stratified by adjustment for stroke


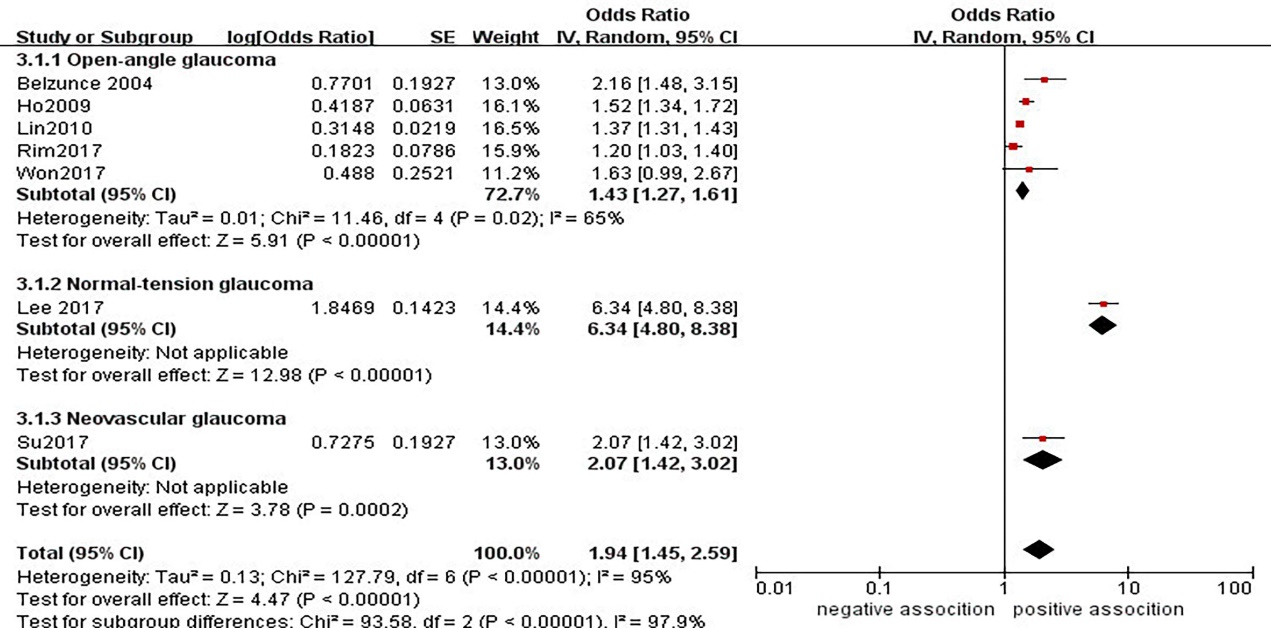


**Figure S3**. Forest plot of subgroup analysis stratified by adjustment for glaucoma


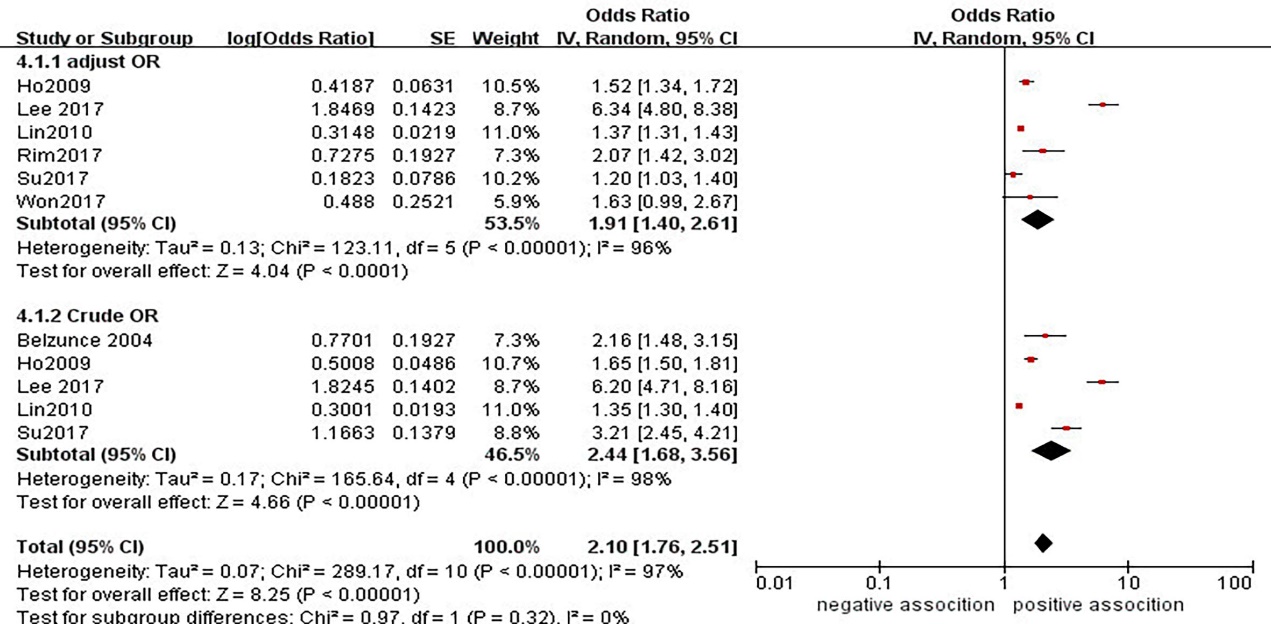


**Figure S4**. Forest plot of subgroup analysis stratified by adjustment for confounders


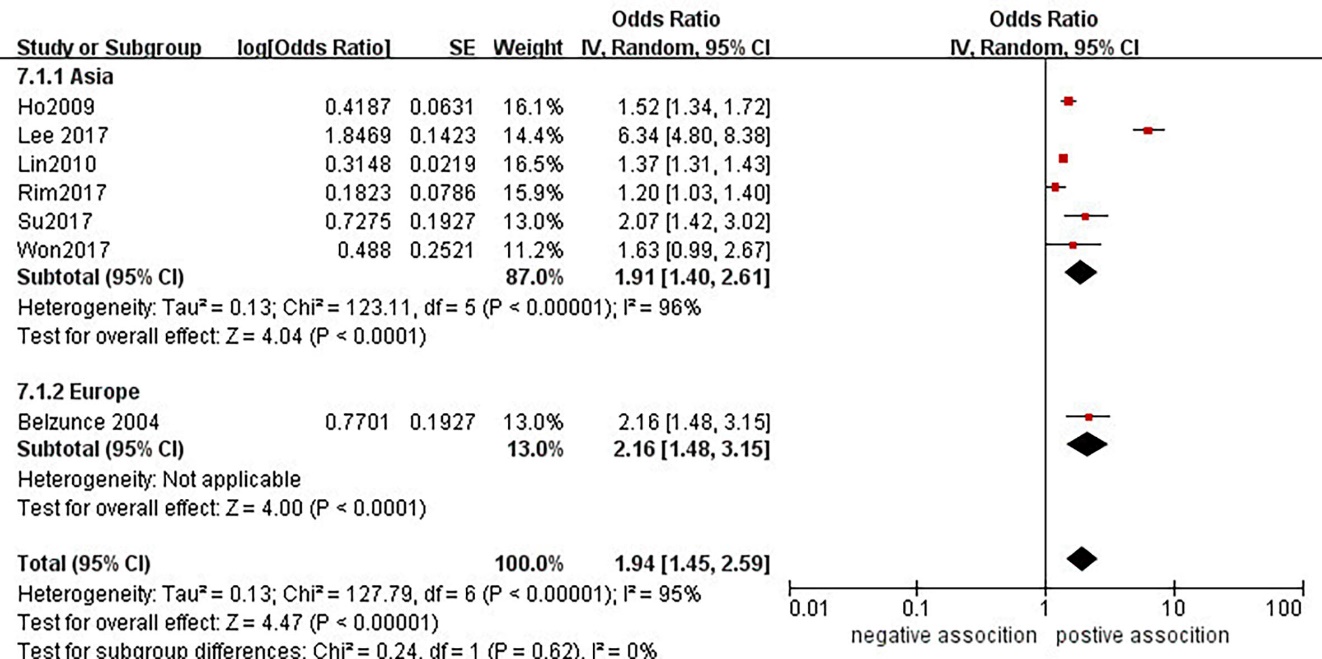


**Figure S5**. Forest plot of subgroup analysis stratified by adjustment for region


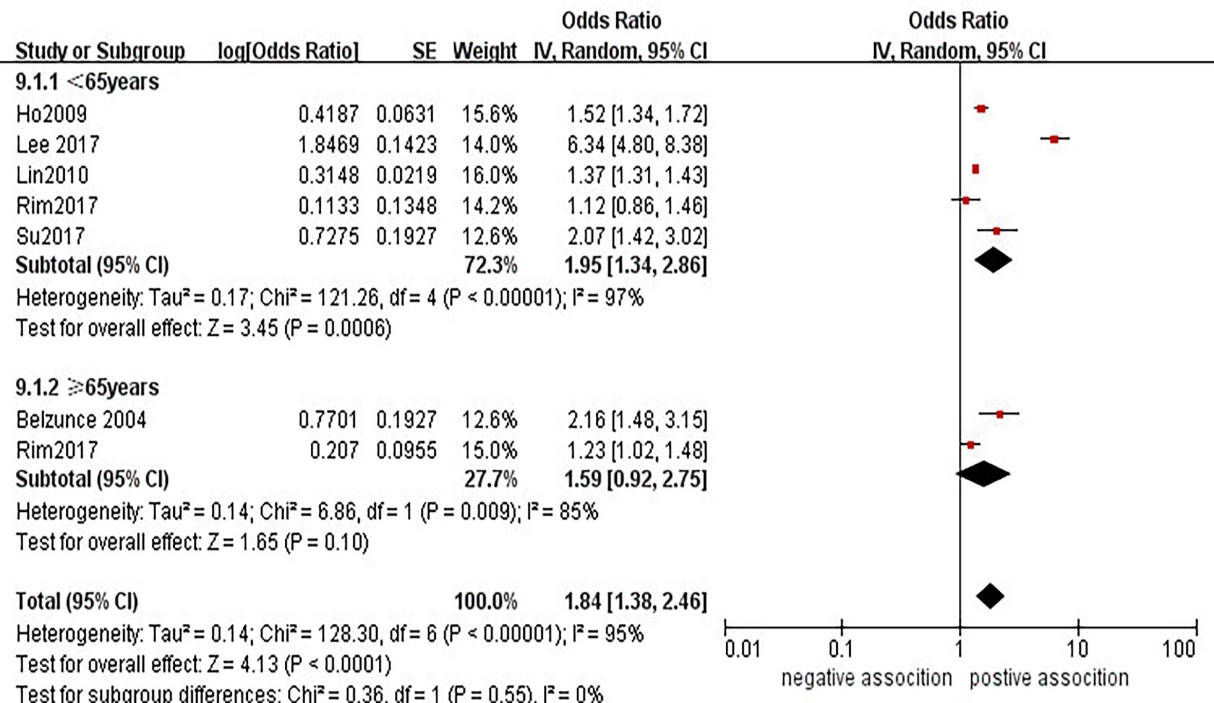


**Figure S6**. Forest plot of subgroup analysis stratified by adjustment for mean age


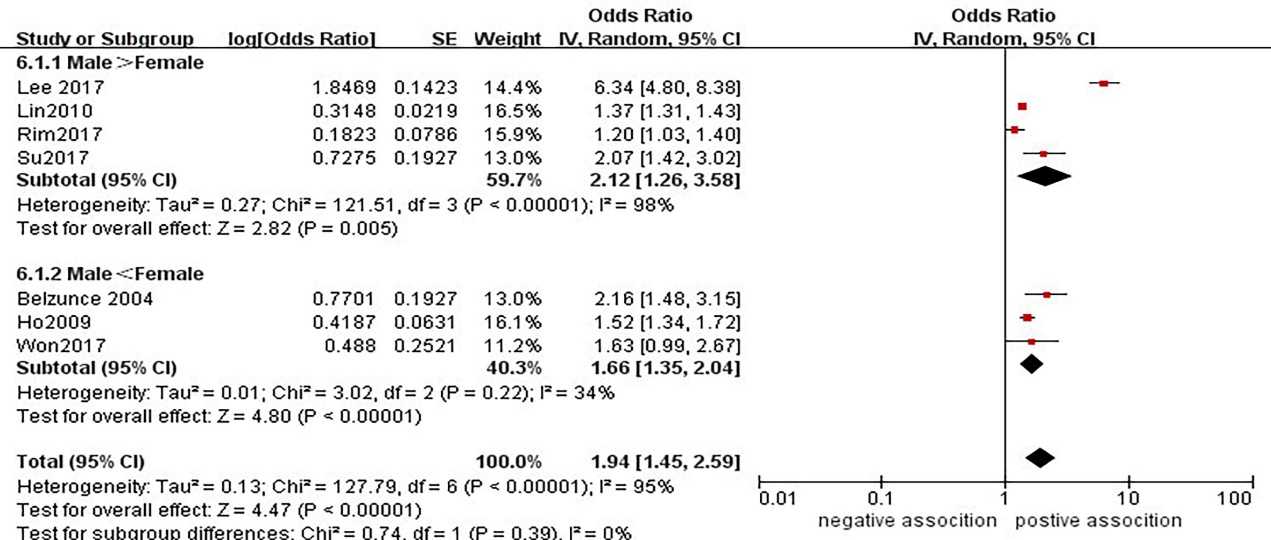


**Figure S7**. Forest plot of subgroup analysis stratified by adjustment for gender


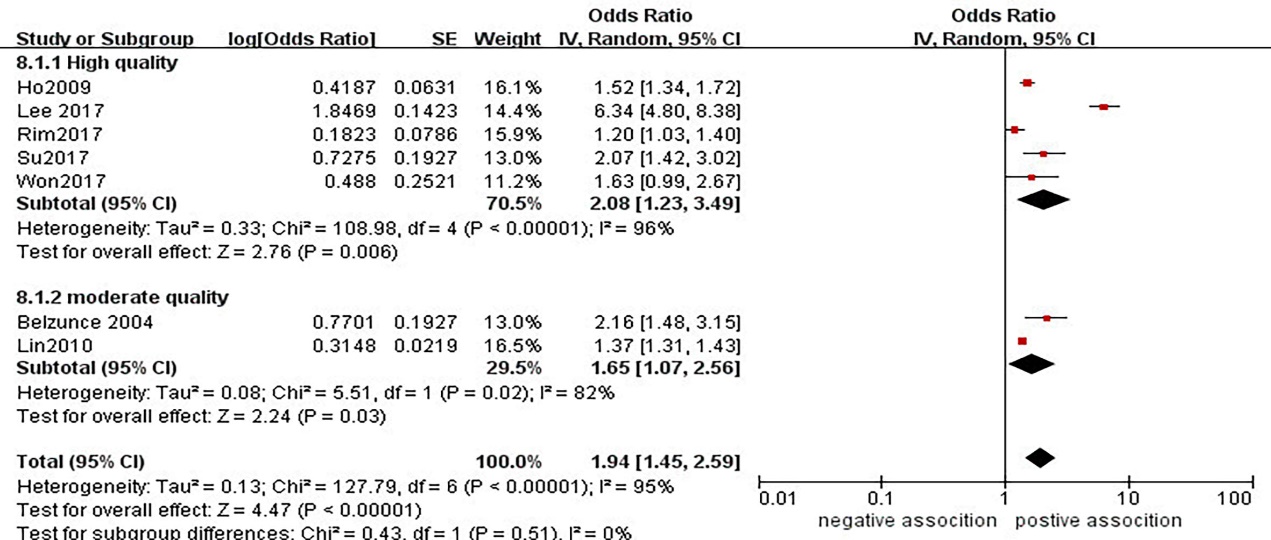


**Figure S8**. Forest plot of subgroup analysis stratified by adjustment for study quality
